# Identification and Validation of a New Set of Five Genes for Prediction of Risk in Early Breast Cancer

**DOI:** 10.3390/ijms14059686

**Published:** 2013-05-06

**Authors:** Giorgio Mustacchi, Maria Pia Sormani, Paolo Bruzzi, Alessandra Gennari, Fabrizio Zanconati, Daniela Bonifacio, Adriana Monzoni, Luca Morandi

**Affiliations:** 1 Cancer Centre, ASS1 University of Trieste, Trieste 34012, Italy; E-Mail: gmustacchi@units.it; 2 Biostatistics Unit, Department of Health Sciences, University of Genova, Genova 16121, Italy; E-Mail: mariapia.sormani@unige.it; 3 Clinical Epidemiology, National Cancer Research Centre, Genova 16132, Italy; E-Mail: paolo.bruzzi@istge.it; 4 Medical Oncology Unit, Galliera Hospital, Genova 16128, Italy; E-Mail: gennari.alessandra@gmail.com; 5 Anatomic Pathology Unit, University of Trieste, Trieste 34010, Italy; E-Mails: fabrizio.zanconati@aots.sanita.fvg.it (F.Z.); d.bonifacio@fmc.units.it (D.B.); 6 Alphagenics Biotechnologies S.r.l., Area Science Park, Basovizza 34012, Italy; E-Mail: adriana.monzoni@alphagenics.it; 7 Department of Biomedical and Neuromuscular Sciences, Section of Anatomic Pathology “M. Malpighi”, Alma Mater Studiorum-University of Bologna, Bologna 40139, Italy

**Keywords:** breast cancer signature, RTqPCR, algorithm, FFPE, prognostic assay

## Abstract

Molecular tests predicting the outcome of breast cancer patients based on gene expression levels can be used to assist in making treatment decisions after consideration of conventional markers. In this study we identified a subset of 20 mRNA differentially regulated in breast cancer analyzing several publicly available array gene expression data using R/Bioconductor package. Using RTqPCR we evaluate 261 consecutive invasive breast cancer cases not selected for age, adjuvant treatment, nodal and estrogen receptor status from paraffin embedded sections. The biological samples dataset was split into a training (137 cases) and a validation set (124 cases). The gene signature was developed on the training set and a multivariate stepwise Cox analysis selected five genes independently associated with DFS: *FGF18* (HR = 1.13, *p* = 0.05), *BCL2* (HR = 0.57, *p* = 0.001), *PRC1* (HR = 1.51, *p* = 0.001), *MMP9* (HR = 1.11, *p* = 0.08), *SERF1a* (HR = 0.83, *p* = 0.007). These five genes were combined into a linear score (signature) weighted according to the coefficients of the Cox model, as: 0.125*FGF18* − 0.560*BCL2* + 0.409*PRC1* + 0.104*MMP9* − 0.188*SERF1A* (HR = 2.7, 95% CI = 1.9–4.0, *p* < 0.001). The signature was then evaluated on the validation set assessing the discrimination ability by a Kaplan Meier analysis, using the same cut offs classifying patients at low, intermediate or high risk of disease relapse as defined on the training set (*p* < 0.001). Our signature, after a further clinical validation, could be proposed as prognostic signature for disease free survival in breast cancer patients where the indication for adjuvant chemotherapy added to endocrine treatment is uncertain.

## 1. Introduction

In the last few years, several multi-gene assays performed on tumor tissue from women with early breast cancer have been proposed to provide prognostic information and discriminate good *vs.* poor prognosis [1–15]. These assays might be useful to assist in making more informed treatment decisions regarding chemotherapy, according to the main international guidelines [16,17].

The array gene expression analysis “Mammaprint®” identifies a 70 gene-signature indicative for poor prognosis in patients with lymph node-negative disease or with 1–3 positive nodes, predicting chemotherapy benefit in the “high risk” group, *vs.* no apparent benefit in the “low risk” group [3–6], in a non-randomized clinical setting. It needs fresh/frozen tissue of the primary breast tumors [2,3]. The multigene assay “Oncotype DX^®^” evaluate gene expression analysis of 21 genes starting from paraffin-embedded tissue calculating a recurrence score to classify patients at low, intermediate, or high risk for recurrence. From two independent retrospective analyses from phase III clinical trial with adjuvant tamoxifen-alone control arms, the 21-gene recurrent score (RS) assay defines a group of patients with low scores who do not appear to benefit from chemotherapy, and a second group with very high scores who derive major benefit from chemotherapy, independently of age and tumor size [1,9–11].

Other studies using a supervised approach based on clinical outcome endpoint to tumor grade as a basis for gene findings have resulted in development of multiple commercial reference lab assays for prognostication (MapQuant Dx [14], Theros Breast Cancer Index [15]).

The above-mentioned multigene assays are expensive and validations have been made on patients selected by age and nodal or Estrogen Receptor status and or received adjuvant treatment.

Analyzing data from several array based gene expression wide analysis publicly available on NCBI Gene Expression Omnibus (GEO; http://www.ncbi.nlm.nih.gov/geo/), we identified a subset of 20 mRNA differentially regulated in breast cancer. We activated a protocol evaluating these markers to create a new gene signature based on real time PCR from paraffin embedded tissue and on a “real life” breast cancer patient population. The enrolled cases were not selected for age, adjuvant treatment, nodal and estrogen receptor status.

## 2. Results and Discussion

**Figure 1:**
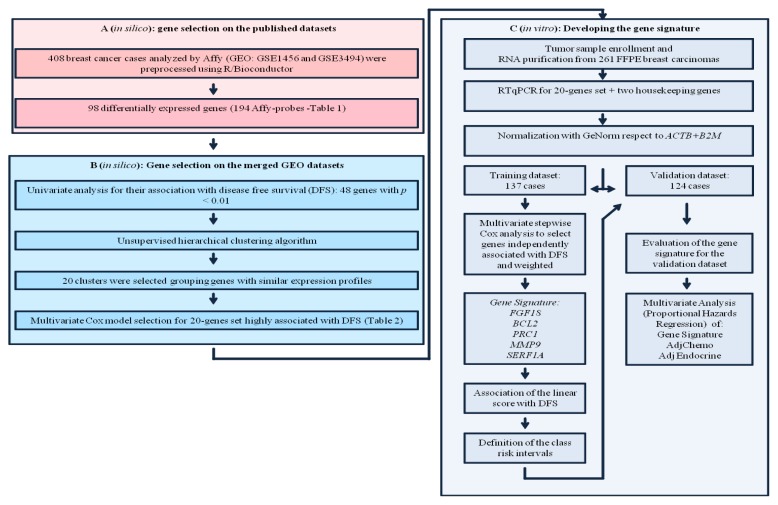
Construction of the gene-set predictor/gene signature for risk prediction. (**A**) Gene selection on the published datasets; (**B**) Gene selection on the merged Gene Expression Omnibus (GEO) datasets; (**C**) Developing the gene signature.

Formalin-fixed and paraffin-embedded (FFPE) tissues represent one of the largest tissue sources, for which well-documented clinical follow-up is available, and therefore large-scale retrospective studies are possible [18]. As described recently by Bussolati *et al.* [19], in a near future the possibility of obtaining high-quality total RNA from archival tissues will guarantee a more powerful and robust gene expression analysis. In order to identify a small number of informative genes providing prognostic information for breast cancer, we evaluated *in silico* a set of published signatures and tested by gene expression array on the 408 breast cancer cases deposited in NCBI Gene Expression Omnibus. By several steps involving univariate analysis for the association with disease free survival (DFS), unsupervised hierarchical clustering algorithm, and multivariate Cox modelling selection, we found 20 highly related genes with DFS. These candidate genes were subsequently evaluated *in vitro* by RTqPCR analyzing a total of 261 cases representing the training (137 cases) and the validation (124 cases) datasets (see the workflow shown in Figure 1).

### 2.1. Gene Selection on the Published Datasets

We used data deposited in NCBI Gene Expression Omnibus (GEO; http://www.ncbi.nlm.nih.gov/geo/, GEO Series accession number GSE1456 and GSE3494), including 408 breast cancer cases. Files containing raw intensity data of Affymetrix HU133A and HU133B arrays of the two datasets (GSE1456 and GSE3494) were preprocessed using R/Bioconductor (GCRMA package, quantile normalization, median polish summarization). The two data sets were pre-processed together using the supercomputer Michelangelo (http://www.litbio.org). The candidate genes were selected from the above mentioned datasets as those included in 4 previously proposed signatures: the “70-gene signature” developed by van de Vijver *et al.* [3] and van’t Veer *et al.* [2] including 70 genes, the “recurrence-score” developed by Paik *et al.* [9] including 21 genes, the “two-gene-ratio model” [12] including 2 genes and the “Insulin Resistance” signature including 15 genes [20] (Table 1). Since some genes are present in more than one signature, the final extracted set was made up of 98 genes (194 Affy-probes) (Table 1).

### 2.2. Gene Selection on the Merged GEO Datasets

The 98 genes selected from the published signatures were first tested in univariate analysis for their association with disease free survival (DFS). Forty-eight genes resulted associated with DFS with a *p* value < 0.01 and were selected for the subsequent step. Using an unsupervised hierarchical clustering algorithm, 20 clusters were selected grouping genes with similar expression profiles. A gene was selected within each cluster using a multivariate Cox model, choosing the one most associated with DFS: the final 20-genes set, all highly associated with DFS, are reported in Table 2.

### 2.3. Tumor Samples

Among 350 consecutive invasive breast cancer patients with full information about tumor, adjuvant treatments, follow up, relapse, death and causes of death, treated between 1998 and 2001, 89 cases (25.4%) were removed from the study because of the low RNA concentration (below 10 ng/μL) or high degradation (*Ct* values for *ACTB* and *B2M* over 34). The remaining 261 cases were split in two biological sample datasets: The training (137 cases) and the validation set (124 cases) by a simple criteria of consecutiveness.

The clinical and demographic characteristics of the patients included in the training and in the validation set are summarized in Table 3 and reported in detail in the supplementary file. Due to a simple criteria of consecutiveness building the sets, the Training set has a longer mean follow up (100.7 months; range 59–123) as compared with the Validation set (89.2; 61–121). Nevertheless, the only significant differences between the two sets was the use of anthracycline-based regimens in the adjuvant setting (Training 16% *vs.* Validation 32.2%; *p* = 0.01) and an higher incidence of G3 tumors in the Validation Set (30.6% *vs.* 19.7, *p* = 0.04). The lack of information about HER2 Status is related to the temporal context of the selected cases (1998–2001) and it was evaluated “*a posteriori*” just in 40% of relapsed patients. Any other clinical and biological pattern is similar and reflecting the “real life” picture of the disease in North East of Italy at this time.

### 2.4. Signature Definition on the Training Set

A multivariate stepwise Cox analysis was run on the breast cancer samples including the 20 selected genes. The Cox model selected a final set of five genes independently associated with DFS (Table 4): *FGF18* (HR = 1.13, *p* = 0.05), *BCL2* (HR = 0.57, *p* = 0.001), *PRC1* (HR = 1.51, *p* = 0.001), *MMP9* (HR = 1.11, *p* = 0.08), *SERF1a* (HR = 0.83, *p* = 0.007).

These five genes were combined into a linear score (signature) weighted according to the coefficients of the Cox model (Table 4), as:

(1) Genesignature=0.125×FGF18-0.560×BCL2+0.409×PRC1+0.104×MMP9-0.188×SERF1

This score ranged from −2.95 to 2.91, with a mean value of −0.48 a SD of 1.00. The linear score was highly associated with DFS in the training set: HR = 2.7, 95% CI = 1.9–4.0, *p* < 0.001.

**Figure 2:**
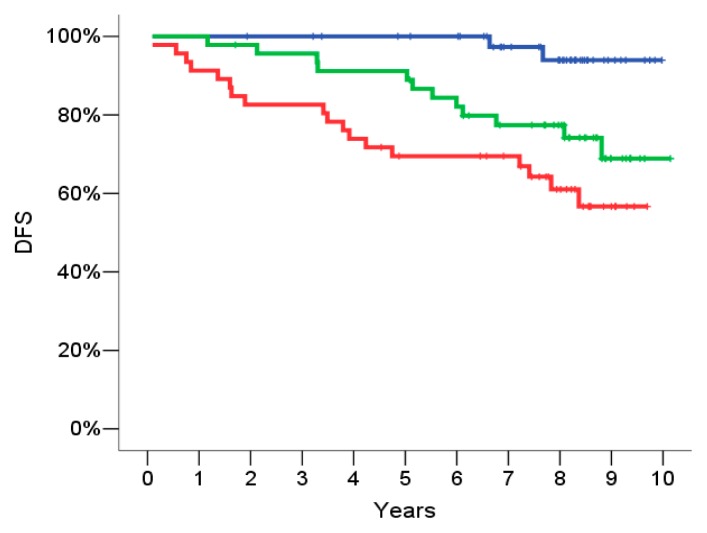
Training set: Probability of 5 years relapse: Disease free survival (DFS) according to the risk groups defined by the gene signature in the training set: Low risk group (blue curve), intermediate risk group (green curve), high risk group (red curve). The hazard ratio (HR) of DFS for intermediate risk patients as compared to low risk is 6.0 (95% Confidence Intervals (CI) = 1.35–27.0, *p* = 0.019 and the HR of DFS for high risk patients as compared to low risk is 10.8 (95% CI = 2.51–46.6, *p* = 0.001).

The score was then categorized in three groups according to the tertiles of its distribution. The DFS according to the three risk groups is reported in Figure 2: Patients with an intermediate risk signature had an HR = 6.03, (95% CI = 1.35–27.0, *p* = 0.019) and patients with a high risk signature had an HR = 10.8, (95% CI = 2.51–46.64, *p* = 0.001) as compared to patients with a low risk signature.

### 2.5. Signature Evaluation on the Validation Set

The signature defined on the training set was evaluated on the independent set of data of the 124 patients included in the validation set. The discrimination ability of the signature was assessed on the validation set by a Kaplan Meier analysis, using the same cut offs classifying patients at low, intermediate or high risk of disease relapse as defined on the training set.

**Figure 3:**
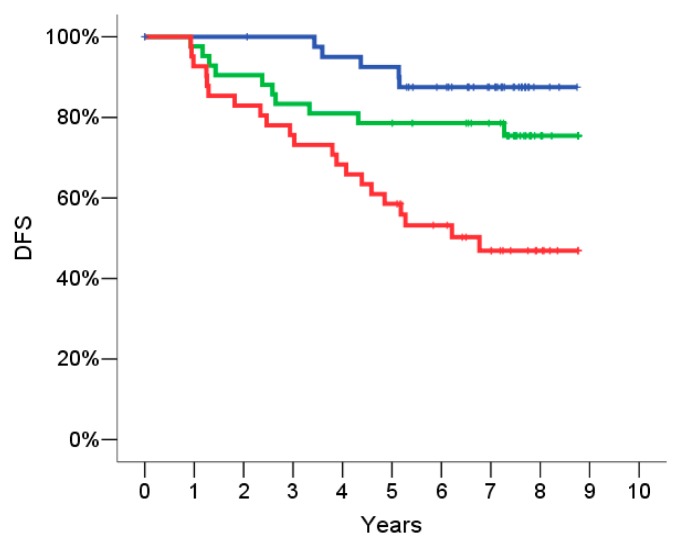
Validation set: Probability of 5 years relapse. Disease free survival (DFS) according to the risk groups defined by the gene signature in the validation set: low risk group (blue curve), intermediate risk group (green curve), high risk group (red curve). The hazard ratio (HR) of DFS for intermediate risk patients as compared to low risk is 2.1 (95% Confidence Intervals (CI) = 0.72–6.2, *p* = 0.17) and the HR of DFS for high risk patients as compared to low risk is 5.4 (95% CI = 2.0–14.4, *p* = 0.001).

The score resulted highly associated with DFS also in the validation set (*p* < 0.001) (Figure 3). Patients with an “intermediate risk” signature had an HR = 2.1 (95% CI = 0.72–6.2, *p* = 0.17) and patients with a high risk signature had an HR = 5.4 (95% CI = 2.0–14.4, *p* = 0.001) as compared to patients with a low risk signature.

### 2.6. Inter and Intra Assay Reproducibility

Three serial sections from three cases each were evaluated independently in triplicate calculating the coefficients of variation (CVs) for the Recurrent Score in the same run and in different runs. The intra-assay and the inter-assay CVs was 3.7% and 4.7%, respectively.

### 2.7. Univariate Analysis

In the Univariate Analysis variables significantly related to DFS were Nodal Status (*p* = 0.0000001), T Size (*p* = 0.000002), the five gene Signature (*i* = 0.000043), Ki67 (*p* = 0.0007) and Grading (*p* = 0.027) (Table 5).

### 2.8. Multivariate Analysis

The Multivariate Analysis (Cox Regression) indicates that Nodal Status (*p* = 0.00001), T Size (*p* = 0.0002) and the five-gene Signature (*p* = 0.0004) are significantly related to DFS, while Ki67 (cut off: 14%), Grading and Chemo- or Endocrine Adjuvant Treatments are not (Table 6). The five-gene Signature HR is slightly affected by adjuvant treatments: Table 7 summarized data about the five-gene signature in presence or absence of Adjuvant treatment.

### 2.9. Discussion

In this study we developed a five-gene recurrence score able to estimate the likelihood of recurrence in a series of consecutive breast cancer tissue samples. These five informative genes were selected by a multistep approach summarized in Figure 1. Firstly, we identified *in silico* a subset of 20 mRNA differentially regulated in breast cancer analyzing several publicly available array gene expression data using R/Bioconductor package. We further evaluated, *in vitro*, the expression level of these 20 genes in 261 consecutive invasive breast cancer cases not selected for age, adjuvant treatment, nodal and estrogen receptor status from paraffin embedded sections. The only requested feature was a minimum follow up of 5 years with full clinical data. Each tissue block was reviewed by a pathologist to ensure greater than 70% content of tumor cells. The gene expression analysis was based on RTqPCR. The biological samples dataset was split into a training and a validation dataset. The gene signature was developed on the training set by a multivariate stepwise Cox analysis selecting five genes independently associated with DFS. These five genes were combined into a linear score (signature) weighted according to the coefficients of the Cox model. The signature was then evaluated on the validation set assessing the discrimination ability by a Kaplan Meier analysis, using the same cut offs classifying patients at low, intermediate or high risk of disease relapse as defined on the training set.

These five genes of interest were identified without any *a priori* selection for gene function or cancer involvement, but simply for the relationship between their expression level and DFS. Interestingly, except for *SERF1a* which the function is still unknown, they have been described to play an important role in cancer as follows:

(a) *FGF18*: Its over-expression in tumors has also been demonstrated [21,22]. *FGF18* expression is up-regulated through the constitutive activation of the *Wnt* pathway observed in most colorectal carcinomas [23]. As a secreted protein, FGF18 can thus affect both the tumor and the connective tissue cells of the tumor microenvironment.
(b) *BCL2*: Over-expression of BCL2 protein has been identified in a variety of solid organ malignancies, including breast cancer. *BCL2* transcript over-expression is related to unfavorable prognosis in Oncotype Dx [9] and in Mammaprint® [3].
(c) *PRC1*: It associates with the mitotic spindle and has been found to play a crucial role in the completion of cytokinesis [24,25]. *PRC1* is negatively regulated by p53 and it is over-expressed in p53 defective cells [26] suggesting that the gene is tightly regulated in a cancer-specific manner.
(d) *MMP9*: Metalloproteases are frequently up-regulated in the tumor microenvironment [27]. *MMP9* influence many aspects of tissue function by cleaving a diverse range of extracellular matrix, cell adhesion, and cell surface receptors, and regulate the bioavailability of many growth factors and chemokines [28].
(e) *SERF1a*: The function of *SERF1a* is not already known.

The biological properties of these genes are related with four of the six hallmarks of cancer proposed by Hanahan *et al.* [29,30]: *FGF18* should be included in “Self-sufficiency in growth signal” group, *BCL2* in “Evading apoptosis” group, *PRC1* in “Limitless replication potential” group, *MMP9* in “Tissue invasion and metastasis” group, while the function of *SERF1a* is still unknown. These findings establish a link between our proposed molecular signature of breast cancer and the underlying capabilities acquired during the multistep development of human tumors previously categorized [29,30].

For an experimental point of view, our assay appears affordable, not time consuming, it needs FFPE tissue and it might be performed easily in almost all laboratories with the required RT-qPCR instrumentations. Importantly it was validated on a “real life” clinical setting with a set of consecutive breast cancer cases irrespectively from age, nodal and estrogen receptor status, adjuvant treatment with at least a minimum follow up of 5 years. An important limit of our approach was that the test was possible in 74.6% of the initial set of cases due to RNA degradation from FFPE tissues according to the literature regarding other signatures [19,31,32]. RNA degradation can be monitored simply evaluating the Ct values of the housekeeping genes used for normalization. Multicentric studies will be needed to evaluate possible pitfalls due to experimental inter-laboratory variability and above all increasing the reliability of the assay. A further step will be the analysis of the predictive value of the five-gene signature in ER positive population of tamoxifen alone benefit and of chemotherapy added to tamoxifen.

## 3. Experimental Section

### 3.1. Tumor Samples Enrolled in This Study

Tumor samples were obtained from routinely processed formalin-fixed, paraffin embedded sections retrieved from 350 consecutive invasive breast cancer patients with full information about tumor, adjuvant treatments, follow up, relapse, death and causes of death, treated between 1998 and 2001. In order to test our signature in a “real life” clinical setting, we decided to use consecutive non metastatic breast cancer cases irrespectively from age, nodal and estrogen receptor status, adjuvant treatment. The only requested pattern was a minimum follow up of 5 years with full clinical data. All patient information was handled in accordance with review board approved protocols and in compliance with the Helsinki declaration [33]. Hematoxylin and Eosin (H & E) sections were reviewed to identify paraffin blocks with tumor areas. Histological type and grade were assessed according to the World Health Organization criteria [34]. The detailed histological and clinical feature of each patient enrolled in this study is available in the supplementary information file. Paraffin blocks corresponding to histology sections that showed the highest relative amount of tumor *vs.* stroma, few infiltrating lymphoid cells and that lacked significant areas of necrosis were selected. Three 20 μm thick sections were cut, followed by one H & E control slide. The tumor area selected for the analysis was marked on this control slide to ensure greater than 70% content of neoplastic cells. Tumor areas dissected ranged from 0.5 to 1.0 cm^2^ wide.

### 3.2. Ethics Statement

The use of tissues for this study has been approved by the Ethics Committee of Centro Oncologico, ASS1 triestina & Università di Trieste, Italy. A comprehensive written informed consent was signed for the surgical treatment that produced the tissue samples and the related diagnostic procedures. All information regarding the human material used in this study was managed using anonymous numerical codes, clinical data were not used and samples were handled in compliance with the Helsinki declaration (http://www.wma.net/en/30publications/10policies/b3/).

### 3.3. Gene Expression Analysis on Breast Cancer Samples

#### 3.3.1. RNA Isolation

Paraffin-embedded tumor material obtained from the 20 μm thick sections was de-paraffinized in xilene at 50 °C for 3 min and rinsed twice in absolute ethanol at room temperature. Total RNA was extracted using the RecoverAll kit (Ambion, Austin, TX, USA), including a DNase step according to the manufacturer’s recommended protocol. RNA concentration was measured by Quant-iT™ RNA kit (Invitrogen, Carlsbad, CA, USA).

#### 3.3.2. Primers Design

Primers were designed using Primer3 software (http://simgene.com/Primer3) and are described in Table 8. Amplicons were tested by *MFOLD* (http://mfold.rna.albany.edu/?q=mfold) in order to avoid secondary structures within primer positions and they were tested by repeatmasker (http://www.repeatmasker.org) and primer-BLAST (http://www.ncbi.nlm.nih.gov/tools/primer-blast) for primer specificity.

#### 3.3.3. Two Step RTqPCR Analysis

Fourteen μL of total RNA was subjected to reverse transcription using SuperScript® VILO™ cDNA Synthesis kit (Invitrogen, Carlsbad, CA, USA) according to the manufacturer’s recommended protocol. One microlitres of cDNA was amplified in duplicate adding 10 picomoles of each primer (see Table 8 for sequence details) to the 1x QuantiFast™ SYBR® Green PCR solution (Qiagen, Hilden, Germany) in a final volume of 25 μL.

Cycling conditions consisted of 5 min at 95 °C, 10 s at 95 °C, 30 s at 60 °C for a total of 40 cycles, using Stratagene Mx3000™ or ABI SDS 7000™ instruments. Plate reading was performed during the 60 °C step.

For each primer set, standard curves made from serial dilutions of cDNA from MCF7 cell lines (see Table 2) were used to estimate PCR reaction efficiency (E) using the formula: E (%) = (10^ [−1/slope]^ − 1) × 100. The expression levels of each of the 20 genes selected were normalized by *GeNorm* [35] using 2 housekeeping genes (*B2M* e *ACTB*) and the relative quantification was calculated by the statistical computing language *R*. The human breast cancer cell line MCF7 was purchased from American Type Culture Collection (ATCC HTB22; derived from a human breast adenocarcinoma). Cells were maintained in minimal essential medium (MEM) (Invitrogen/Life technologies, Villebon-sur-Yvette, France) supplemented with 2 mM l-glutamine, 1.5 g/L sodium bicarbonate, 0.1 mM nonessential aa, 1 mM pyruvate sodium, 0.01 mg/mL bovine insulin, and 10% fetal bovine serum (Thermo Scientific, Waltham, MA, USA) at 37 °C in a humidified atmosphere of 5% CO_2_.

### 3.4. Training and Validation Dataset

The biological samples dataset was split into the training and the validation dataset. The training set consists of the first 144 consecutive cases and the validation of the last 127 cases. The gene signature was developed on the training set. Once the signature has been fully specified, the validation set was accessed once and only for estimating the prediction accuracy of the signature. A multivariate stepwise Cox analysis was run on the breast cancer training set samples including the 20 selected genes. The stepwise procedure was run to select genes independently associated with DFS (*p* for inclusion <0.10). The overall workflow shown in Figure 1 summarizes every step starting from selection of markers from the literature since the validation of the gene signature. Reproducibility within and between blocks was assessed by performing the test in serial sections from three blocks representing three cases. We finally performed a multivariate Cox proportional-hazards analysis in a model that included treatment received (no adjuvant therapy *vs.* chemotherapy, hormonal therapy, or both) and the final gene Signature (both Training and Validation sets included), using the NCSS 2001 Statistical software (NCSS Inc., Kaysville, UT, USA, 2001).

### 3.5. Univariate and Multivariate Analysis

We performed a univariate analysis including Age, T size, Nodal status, Grading, Ki67, adjuvant treatments and the 5-gene signature, followed by a multivariate Cox proportional-hazards analysis in a model that included treatment received (no adjuvant therapy *vs.* chemotherapy, hormonal therapy, or both) and the 5-gene Signature (Low/Intermediate/High Risk; both Training and Validation sets included), using the NCSS 2001 Statistical software (NCSS Inc., Kaysville, UT, USA, 2001).

## 4. Conclusions

We developed a prognostic tool for early breast cancer based on the analysis of the relative expression level of *FGF18*, *BCL2*, *PRC1*, *MMP9* and *SERF1A* in combination. Our signature has a good discriminating ability when tested on the validation set. We suppose that, after a necessary further clinical validation on a higher number of cases, it could be proposed as non expensive prognostic signature for disease free survival in breast cancer patients where the indication for adjuvant chemotherapy added to endocrine treatment is uncertain.

## Figures and Tables

**Table 1:** Genes selected and also present in other previously published signatures (1 = van’t Veer *et al.* [2], 2 = Paik *et al.* [9], 3 = Gennari *et al.* [20], 4 = 2 Ma *et al.* [12], 1.5 = van’t Veer *et al.* [2] with Paik *et al.* [9]).

Symbol	AffyID	Group	Affychip	Symbol	AffyID	Group	Affychip	Symbol	AffyID	Group	Affychip
ALDH4A1	203722_at	1.00	A	MKI67	212021_s_at	2.00	A	MCM6	201930_at	1.00	A
AP2B1	200612_s_at	1.00	A	MKI67	212022_s_at	2.00	A	MELK	204825_at	1.00	A
AP2B1	200615_s_at	1.00	A	MKI67	212023_s_at	2.00	A	MKI67	212020_s_at	2.00	A
AURKA	204092_s_at	2.00	A	MMP11	203876_s_at	2.00	A	SLC2A3	240055_at	1.00	
AURKA	208079_s_at	2.00	A	MMP11	203878_s_at	2.00	A	ZNF533	229019_at	1.00	
AURKA	208080_at	2.00	A	MMP9	203936_s_at	1.00	A	ZNF533	243929_at	1.00	
AYTL2	201818_at	1.00	A	MYBL2	201710_at	2.00	A	IGF1	209540_at	3.00	A
BAG1	202387_at	2.00	A	NDC80	204162_at	1.00	A	IGF1R	203628_at	3.00	A
BAG1	211475_s_at	2.00	A	NUSAP1	218039_at	1.00	A	IGF2	202410_x_at	3.00	A
BBC3	211692_s_at	1.00	A	ORC6L	219105_x_at	1.00	A	IGFBP4	201508_at	3.00	A
BC045642	212248_at	1.00	A	OXCT1	202780_at	1.00	A	IGFBP5	203424_s_at	1.00	A
BC045642	212250_at	1.00	A	PALM2-AKAP2	202759_s_at	1.00	A	IGFBP5	203425_s_at	1.00	A
BC045642	212251_at	1.00	A	PALM2-AKAP2	202760_s_at	1.00	A	IGFBP5	203426_s_at	1.00	A
BCL2	203684_s_at	2.00	A	PECI	218025_s_at	1.00	A	IGFBP5	211958_at	1.00	A
BCL2	203685_at	2.00	A	PGR	208305_at	2.00	A	IGFBP5	211959_at	1.00	A
BCL2	207004_at	2.00	A	PITRM1	205273_s_at	1.00	A	IGFBP6	203851_at	3.00	A
BCL2	207005_s_at	2.00	A	PQLC2	220453_at	1.00	A	IGFBP7	201163_s_at	3.00	A
BF034907	206023_at	1.00	A	PRC1	218009_s_at	1.00	A	IL17RB	219255_x_at	4.00	A
BIRC5	202094_at	2.00	A	RAB6A	201045_s_at	1.00	A	IL6ST	204863_s_at	3.00	A
BIRC5	202095_s_at	2.00	A	RAB6A	201047_x_at	1.00	A	INSIG1	201627_s_at	3.00	A
BIRC5	210334_x_at	2.00	A	RAB6A	201048_x_at	1.00	A	IRS1	204686_at	3.00	A
C16orf61	218447_at	1.00	A	RAB6A	210406_s_at	1.00	A	IRS2	209184_s_at	3.00	A
C20orf46	219958_at	1.00	A	RFC4	204023_at	1.00	A	LGP2	219364_at	1.00	A
C9orf30	205122_at	1.00	A	SCUBE2	219197_s_at	1.50	A	LOC643008	229740_at	1.00	B
C9orf30	205123_s_at	1.00	A	SERF1A	219982_s_at	1.00	A	MCM6	238977_at	1.00	B
CCNB1	214710_s_at	2.00	A	SLC2A3	202497_x_at	1.00	A	MS4A7	223343_at	1.00	B
CCNE2	205034_at	1.00	A	SLC2A3	202498_s_at	1.00	A	MS4A7	223344_s_at	1.00	B
CCNE2	211814_s_at	1.00	A	SLC2A3	202499_s_at	1.00	A	MS4A7	224358_s_at	1.00	B
CD68	203507_at	2.00	A	SLC2A3	216236_s_at	1.00	A	PALM2-AKAP2	226694_at	1.00	B
CDC42BPA	214464_at	1.00	A	SLC2A3	222088_s_at	1.00	A	QSOX2	227146_at	1.00	B
CENPA	204962_s_at	1.00	A	STK32B	219686_at	1.00	A	QSOX2	235239_at	1.00	B
CENPA	210821_x_at	1.00	A	TGFB3	209747_at	1.00	A	RTN4RL1	229097_at	1.00	B
COL4A2	211964_at	1.00	A	TNFRSF10B	209295_at	3.00	A	RTN4RL1	232596_at	1.00	B
COL4A2	211966_at	1.00	A	TNFRSF12A	218368_s_at	3.00	A	RTN4RL1	242102_at	1.00	B
CTSL2	210074_at	2.00	A	TNFRSF21	214581_x_at	3.00	A	RUNDC1	226298_at	1.00	B
DCK	203302_at	1.00	A	TNFSF10	214329_x_at	3.00	A	RUNDC1	235040_at	1.00	B
DIAPH3	220997_s_at	1.00	A	TSPYL5	213122_at	1.00	A	SERF1A	223538_at	1.00	B
DTL	218585_s_at	1.00	A	UCHL5	219960_s_at	1.00	A	SERF1A	223539_s_at	1.00	B
ECT2	219787_s_at	1.00	A	WISP1	206796_at	1.00	A	SLC2A3	236180_at	1.00	B
EGLN1	221497_x_at	1.00	A	WISP1	211312_s_at	1.00	A	SLC2A3	236571_at	1.00	B
ESM1	208394_x_at	1.00	A	AA834945	230365_at	1.00	B	GRB7	210761_s_at	2.00	A
ESR1	205225_at	2.00	A	AA834945	235039_x_at	1.00	B	GSTM1	204418_x_at	2.00	A
ESR1	207672_at	2.00	A	AI224578	235247_at	1.00	B	GSTM1	204550_x_at	2.00	A
ESR1	211233_x_at	2.00	A	AI283268	232579_at	1.00	B	GSTM1	215333_x_at	2.00	A
ESR1	211234_x_at	2.00	A	AP2B1	234064_at	1.00	B	GSTM3	202554_s_at	1.00	A
ESR1	211235_s_at	2.00	A	AW014921	230710_at	1.00	B	HER2	210930_s_at	2.00	A
ESR1	211627_x_at	2.00	A	AW014921	236480_at	1.00	B	HER2	216836_s_at	2.00	A
ESR1	215552_s_at	2.00	A	AYTL2	241511_at	1.00	B	HOXB13	209844_at	4.00	A
ESR1	217163_at	2.00	A	CDCA7	224428_s_at	1.00	B	HRASLS	219983_at	1.00	A
ESR1	217190_x_at	2.00	A	CDCA7	230060_at	1.00	B	HRASLS	219984_s_at	1.00	A
EXT1	201995_at	1.00	A	COL4A2	237624_at	1.00	B	IDE	203328_x_at	3.00	A
EXT1	215206_at	1.00	A	DCK	224115_at	1.00	B	FBXO31	223745_at	1.00	B
FBXO31	219784_at	1.00	A	DTL	222680_s_at	1.00	B	FBXO31	224162_s_at	1.00	B
FBXO31	219785_s_at	1.00	A	EBF4	233032_x_at	1.00	B	FBXO31	236873_at	1.00	B
FBXO31	222352_at	1.00	A	EBF4	233850_s_at	1.00	B	FGF18	231382_at	1.00	B
FGF18	206986_at	1.00	A	ECT2	234992_x_at	1.00	B	FLT1	226497_s_at	1.00	B
FGF18	206987_x_at	1.00	A	ECT2	237241_at	1.00	B	FLT1	226498_at	1.00	B
FGF18	211029_x_at	1.00	A	EGLN1	223045_at	1.00	B	FLT1	232809_s_at	1.00	B
FGF18	211485_s_at	1.00	A	EGLN1	223046_at	1.00	B	GPR180	231871_at	1.00	B
FGF18	214284_s_at	1.00	A	EGLN1	224314_s_at	1.00	B	GPR180	232912_at	1.00	B
FLT1	204406_at	1.00	A	EXT1	232174_at	1.00	B	GSTM3	235867_at	1.00	B
FLT1	210287_s_at	1.00	A	EXT1	234634_at	1.00	B	LOC286052	241370_at	1.00	B
FLT1	222033_s_at	1.00	A	EXT1	237310_at	1.00	B				
GMPS	214431_at	1.00	A	EXT1	239227_at	1.00	B				
GNAZ	204993_at	1.00	A	EXT1	239414_at	1.00	B				
GPR126	213094_at	1.00	A	EXT1	242126_at	1.00	B				

**Table 2:** Final 20 genes set, all highly associated with Disease free survival (DFS).

Index	Symbol	Cluster	AffyID	Group	Chip	logHR	HR	*p* value
114	PRC1	1	218009_s_at	1	A	0.26	1.29	<0.00001
120	ORC6L	16	219105_x_at	1	A	0.36	1.44	0.000201
38	MMP9	14	203936_s_at	1	A	0.14	1.15	0.000607
11	AYTL2	5	201818_at	1	A	0.38	1.46	0.000828
69	TGFB3	3	209747_at	1	A	−0.23	0.79	0.000860
145	SERF1A	19	223539_s_at	1	B	0.36	1.44	0.001192
163	FGF18	8	231382_at	1	B	−0.41	0.67	0.003375
156	QSOX2	18	227146_at	1	B	0.51	1.66	0.003409
143	MS4A7	15	223344_s_at	1	B	−0.16	0.85	0.004351
126	FBXO31	7	219785_s_at	1	A	0.31	1.36	0.004459
164	GPR180	9	231871_at	1	B	0.33	1.39	0.005603
54	PITRM1	17	205273_s_at	1	A	0.26	1.30	0.007143
33	BCL2	6	203685_at	2	A	−0.16	0.85	0.003310
68	IGF1	2	209540_at	3	A	−0.22	0.80	0.000001
35	IGFBP6	2	203851_at	3	A	−0.40	0,67	0.000002
47	IL6ST	12	204863_s_at	3	A	−0.19	0.83	0.000028
45	IRS1	13	204686_at	3	A	−0.19	0.82	0.001258
7	IGFBP7	4	201163_s_at	3	A	−0.41	0.66	0.001529
102	TNFSF10	20	214329_x_at	3	A	−0.20	0.82	0.004448
26	IDE	11	203328_x_at	3	A	0.52	1.68	0.005188

**Table 3:** Characteristics of patients and tumors in the Training and Validation sets.

	Training Set		Validation Set		*p* value
**Nr of Patients**	137		124		ns
**Mean Age (range)**	62.3 (35–87)		61.1 (33–87)		ns
**Mean Follow up (months)**	100.7 (59–123)		89.2 (61–121)		ns
**Histology**	***n***	**%**	***n***	**%**	***p* value**
Ductal	86	62.8	83	66.9	ns
Lobular	26	19	16	12.9	ns
Tubular-Lobular	12	8.8	10	8.5	ns
Medullary/Apocrine	2	1.4	3	2.4	ns
Other	11	8.02	12	9.6	ns

**T Size**					
T1	78	56.9	82	66.1	ns
T2	53	38.7	37	29.8	ns
T3	3	2.2	3	2.4	ns
Tx	3	2.2	2	1.6	ns

**N Status**					
pN0	89	65	75	60.5	ns
pN1a	26	19	26	21	ns
pN+ 4–10	11	8.1	7	5.6	ns
pN+ >10	10	7.3	14	11.3	ns
NX	0				
ER/PgR pos	123	85.4	97	76.38	ns
HER2 NA	125	91.2	79	73.7	*p* = 0.05*

**Grading**					
G1	33	24.1	20	16.1	ns
G2	51	37.2	57	46	ns
G3	27	19.7	38	30.6	*p* = 0.04
G NA	26	19	9	7.3	ns

**Ki67**					
High (>14%)	60	43.8	60	48.4	Ns
Low (<15%)	77	56.2	60	48.4	ns
Adjuvant Chemo	49	35.8	57	46	ns
Anthracycline-based	22	16	40	32.2	*p* = 0.01
Adjuvant endocrine (any)	110	80.3	96	77.4	*p* = 0.01
Relapses	33	24	38	30.6	ns
Mean DFS, months	51.4		47.2		ns
Deaths	33	24	39	31.4	ns

* In the Validation Set HER2 status was evaluated in relapsed patients.

**Table 4:** Genes selected in the five-genes signature. Variables in the Equation.

							95.0% CI for Exp(B)	

gene	B	SE	Wald	df	Sig.	Exp(B)	Lower	Upper
*FGF18*	0.125	0.064	3.736	1	0.053	1.133	0.998	1.285
*BCL2*	−0.56	0.173	10.4444	1	0.001	0.571	0.407	0.802
*PRC1*	0.409	0.12	11.712	1	0.001	1.506	1.191	1.903
*MMP9*	0.104	0.06	3.031	1	0.082	1.109	0.987	1.247
*SERF1A*	−0.188	0.069	7.375	1	0.007	0.828	0.723	0.949

**Table 5:** Univariate analysis.

Variable	Regression coefficient (B)	SE	Exp (B)	Mean	Z-value	Probability level
Nodal Status (pN0/pN1a/pN2)	0.591	0.100	1.806	0.062	5.1	0.0000001
T Size (pT1/pT2/pT3)	3.647	7.639	1.037	20.195	4.77	0.000002
5 gene Signature (High/Intermediate/Low)	0.646	0.158	1.909	1.984	4.09	0.000043
Ki67 (High/Low)	0.427	0.126	1.533	1.933	3.38	0.0007
Grading (G1/G2/G3)	0.298	0.135	1.348	1.798	2.2	0.027

**Table 6:** Multivariate Cox regression analysis.

Variable	Regression coefficient (B) (95% CI)	SE	Exp (B)	Mean	Z-value	Probability level
Nodal Status (pN0/pN1a/pN2)	0.551 (0.350–0.752)	0.102	1.736	0.655	5.379	0.00001
T Size (pT1/pT2/pT3)	0.562 (0.269–0.854)	0.149	1.754	1.449	3.762	0.0002
5 gene Signature (High/Intermediate/Low)	0.666 (0.298–1.034)	0.187	1.947	1.9767	3.549	0.0004
Ki67 (High/Low)	0.27 (−0.028–0.569)	0.152	1.31	1.748	1.77	0.076
Grading (G1/G2/G3)	−0.111 (−0.387–0.164)	0.14	0.894	1.798	−0.792	0.428
AdjChemo (Yes/No)	0.061 (−0.479–0.601)	0.275	1.063	1.604	0.221	0.824
Adj Endocrine (Yes/No)	0.032 (−0.556–0.622)	0.3	1.033	1.209	0.109	0.912

**Table 7:** Hazard Ratio Longrank (Cox-Mantel) for five-gene signature in presence or absence of adjuvant treatments.

Chemo or endocrine adjuvant treatment						

	YES			NO		
5 Gene Score	HR	95% CI	*p* value	HR	95% CI	*p* value
Low *vs*. High	0.35	0.20–0.60	0.0006	0.16	0.08–0.32	0.0001
Low *vs*. Intermediate	0.98	0.45–2.11	0.9	0.29	0.11–0.77	0.0224
Intermediate *vs*. High	0.4	0.23–0.69	0.002	0.56	0.29–1.06	0.089

**Table 8:** Primer sequences, slope, PCR efficiency and RSq of each of the 20 genes + 2 housekeeping genes.

	Primer forward	Primer reverse	Slope	Efficiency	RSq
B2M	ATGAGTATGCCTGCCGTGTGA	GGCATCTTCAAACCTCCATG	−3.051	112.7%	0.992
ACTB	TTGCCGACAGGATGCAGAAGGA	AGGTGGACAGCGAGGCCAGGAT	−3.116	109.4%	0.998
FBX031	GAGGACATCTTCCACGAGCAC	AGGTAGATGCGGCGGTAGGT	−3.293	101.2%	0.995
FGF18	GGTAGTCAAGTCCGGATCAAGG	TCCAGAACCTTCTCGATGAACA	−3.217	104.6%	0.952
BCL2	AGTACCTGAACCGGCACCTG	CAGAGACAGCCAGGAGAAATCA	−3.787	83.7%	0.999
IGFBP7	ATGAAGTAACTGGCTGGGTGCT	TGAAGCCTGTCCTTGGGAAT	−3.043	113.1%	0.997
IDE	AGCCCTTCTCCATGGAAACATA	CAGCTGACTTGGAAGGAGAGGT	−3.149	107.8%	0.998
AYTL2	GTTGCCCTGTCTGTCGTCTG	CTTGAGGATGCAGGACAGGT	−3.057	112.4%	0.989
ORC6L	TGAAGTGCCCCTTGGACAG	CAGGCCCAGTAAACACTCAAAAG	−3.093	110.5%	0.996
MS4A7	CCCTCAAAGAGAGAAACCTGGA	ATCAACAGGCAACACAGGATCT	−3.162	107.1%	0.964
OSOX2	CGTGTTCTCTCTGGAAACTGTTC	GAACGTACCTCCTCATTGTCTGC	−3.236	103.7%	0.998
PITRM1	GGAAAATTCACACAGCAAGACA	AGAGGCCGTACAAGAAGTGGT	−3.192	105.7%	0.997
TGFb3	AACTTCTGCTCAGGCCCTTG	AGGCAGATGCTTCAGGGTTC	−3.216	104.6%	0.998
PRC-1-201	CCGTGTCTCGACTTCCTCCT	CGTTGAGCTCCAGGTTCTCC	−3.092	110.6%	0.991
GPR180	GATTCTACGCCTGCATCCACT	CCCTGCTAAGTTGTGGTGTGAA	−3.076	111.4%	0.996
MMP9	GCAAGCTGGACTCGGTCTT	CCTGTGTACACCCACACCTG	−2.198	185.1%	0.953
IGFBP6	GAATCCAGGCACCTCTACCAC	AGTCCAGATGTCTACGGCATGG	−2.821	126.2%	0.998
IRS1	CAGTTTCCAGAAGCAGCCAGAG	GAGGATTTGCTGAGGTCATTTA	−3.136	108.4%	0.990
IL6ST210	CAGTGGTCACCTCACACTCCTC	TTTGTCATTTGCTTCTATTTCCA	−3.071	111.7%	0.972
IGF1	TATCAGCCCCCATCTACCAAC	TCTTGTTTCCTGCACTCCCTCT	−3.012	102.3%	0.998
TNSF	TCCTCAGAGAGTAGCAGCTCACA	CCTTGATGATTCCCAGGAGTT	−2.628	140.2%	0.759
SERF1A	CCAGGAAATTAGCAAGGGAAAG	CTTGTCTGCATAGACTTCTTCTCA	−2.927	119.6%	0.974

## Abbreviation

H & E: Hematoxylin and eosin
DFS: disease free survival
FFPE: Formalin-fixed and paraffin-embedded
ESMO: European Society for Medical Oncology.
